# Comparative void-volume analysis of psychrophilic and mesophilic enzymes: Structural bioinformatics of psychrophilic enzymes reveals sources of core flexibility

**DOI:** 10.1186/1472-6807-11-42

**Published:** 2011-10-20

**Authors:** Diana I Paredes, Kyle Watters, Derek J Pitman, Christopher Bystroff, Jonathan S Dordick

**Affiliations:** 1 Department of Chemical and Biological Engineering, Rensselaer Polytechnic Institute, Troy, NY, USA; 2 Department of Biology, Rensselaer Polytechnic Institute, Troy, NY, USA; 3 Department of Biomedical Engineering, Center for Biotechnology & Interdisciplinary Studies, Rensselaer Polytechnic Institute, Troy, NY, USA

## Abstract

**Background:**

Psychrophiles, cold-adapted organisms, have adapted to live at low temperatures by using a variety of mechanisms. Their enzymes are active at cold temperatures by being structurally more flexible than mesophilic enzymes. Even though, there are some indications of the possible structural mechanisms by which psychrophilic enzymes are catalytic active at cold temperatures, there is not a generalized structural property common to all psychrophilic enzymes.

**Results:**

We examine twenty homologous enzyme pairs from psychrophiles and mesophiles to investigate flexibility as a key characteristic for cold adaptation. B-factors in protein X-ray structures are one way to measure flexibility. Comparing psychrophilic to mesophilic protein B-factors reveals that psychrophilic enzymes are more flexible in 5-turn and strand secondary structures. Enzyme cavities, identified using CASTp at various probe sizes, indicate that psychrophilic enzymes have larger average cavity sizes at probe radii of 1.4-1.5 Å, sufficient for water molecules. Furthermore, amino acid side chains lining these cavities show an increased frequency of acidic groups in psychrophilic enzymes.

**Conclusions:**

These findings suggest that embedded water molecules may play a significant role in cavity flexibility, and therefore, overall protein flexibility. Thus, our results point to the important role enzyme flexibility plays in adaptation to cold environments.

## Background

Life exists over a wide temperature range, from as low as -15°C to as high as 122°C [1]. On the upper end of the temperature spectrum, thermophiles and hyperthermophiles have been studied extensively by the scientific community, particularly the molecular mechanisms that support protein structure and function at high temperatures. For example, compact and strong hydrophobic packing is typically found in most cores of thermophilic proteins, which increases the energy needed to unfold the protein, making it possible for thermophilic proteins to retain native structure at high temperatures [2]. Indeed, a strong correlation exists between high core packing density and thermostability [2-4]. We are also intrigued by the obverse - organisms that survive optimally at cold temperatures; harsh environments with restricted molecular mobility and reduced reaction kinetics that hinder myriad cellular and biomolecular processes [5-7].

Psychrophiles, "cold-loving" microorganisms, have adapted to life at low temperatures by using a variety of mechanisms. These include the production of anti-freeze and cold-shock proteins, alterations in membrane composition, and overexpression of proteins that destabilize DNA structures, among other mechanisms [8-10]. Some of the more interesting adaptations are found in psychrophilic enzymes. In particular, psychrophilic enzymes typically have a higher occurrence of nonpolar residues on their surface, which destabilizes water structure around the enzyme such that water properties are considerably distinct from bulk water, and a lower abundance of arginine and proline residues, which increases backbone flexibility. Moreover, fewer weak interactions, such as hydrogen bonds, reduce the degree of packing within the enzyme core and make psychrophilic enzymes more conformationally flexible [[11-13]; and references therein]. Increased flexibility has been proposed as a major molecular mechanism for the evolution of cold adapted enzymes, a hypothesis supported by spectroscopic analysis (e.g., nuclear magnetic resonance [14], dynamic fluorescence quenching [15] and molecular dynamics (MD) simulations [16]).

Psychrophilic enzymes have been shown to lose more entropy upon transition state activation than their mesophilic counterparts, suggesting that the psychrophilic proteins exist in a more disordered ground state [17,9]. This intuitively makes sense, since in order for psychrophilic enzymes to be more active at low temperatures they must have more kinetic energy [15]. Nonetheless, the structural basis of this flexibility remains unclear. For example, an MD simulations study of one-on-one comparisons of trypsin from Atlantic salmon and its mesophilic counterpart [18] found that the psychrophilic trypsin possesses higher flexibility close to the active site parts. Interestingly, the active site is where there is the most significant difference in amino acid composition between mesophilic and psychrophilic trypsins. However, other psychrophilic enzymes exhibit regions of flexibility in places distant from the active site. For example, psychrophilic uracil-DNA glycosylases [19] have fewer strong ion-pair interactions near the C-terminus than their mesophilic counterparts. This local difference results in increased flexibility in the psychrophilic enzymes. Psychrophilic alpha-amylases [20] show greater overall flexibility than the mesophilic/thermophilic counterparts. This is consistent with thermal unfolding experiments, where the psychrophilic enzymes unfold at low temperatures cooperatively without intermediates due to fewer stabilizing interactions, while thermophilic amylases show intermediates during unfolding, indicating that there are regions with greater rigidity than others. The different strategies that psychrophilic enzymes use to adapt to colder environments have resulted in a number of divergent viewpoints on the influence of local and global protein flexibility on cold adaptation.

An interesting structural property of proteins, particularly as it relates to thermostability, is cavity number and size. Protein thermostability appears to diminish when cavities are created in the protein [21]. Such cavities represent packing defects in the protein core. In the case of psychrophilic proteins, only a few studies have addressed these cavities, and then only with a small set of proteins [11,22]. No clear relationship was observed between psychrophily and total void-volume in the enzyme core, as might be expected from the strong hydrophobic cores present in thermophilic proteins. However, an increased number of three-dimensional structures of psychrophilic proteins have become available and offer the opportunity to revisit such a structural analysis. Here we test the hypothesis that cavity properties in psychrophilic enzymes endow these proteins with the increased conformationally flexibility necessary to function optimally at low temperatures.

In the current work we investigated differences in the average cavity volume and crystallographic waters between psychrophilic and mesophilic homologs. A non-redundant set of 20 psychrophilic enzymes was examined, with each paired to a homologous mesophilic enzyme (sequence identity above 35% to 76%), and all with high resolution crystal structures. In addition to counting cavities and calculating void volumes, we evaluated amino acid frequencies in residue positions surrounding cavities. We present evidence that the average cavity size of psychrophilic enzymes is larger, and contains more surrounding hydrophilic groups, than their mesophilic counterparts. These findings support a hypothesis that psychrophilic enzymes may have a predisposition to having more water molecules within their cavities, which consequently increases enzyme conformational dynamics, leading to greater activity under the rigidifying cold environment. The results provide a potential strategy by which optimal temperature could be altered.

## Results and Discussion

### Database construction

A database of psychrophilic and mesophilic homologous proteins was constructed from several literature references and an in-house pipeline script (Table 1). All 12 psychrophilic enzymes from Siddiqui et al. [12] were included. Additionally, eight psychrophilic enzymes were included from the NCBI database through query using the NCBI entrez tool. A total of 148 mesophilic proteins that were homologous to the 20 psychrophilic proteins were then obtained with DaliLite. The mesophilic proteins were then rejected if they had resolution > 2.5 Å, did not belong to the same enzyme family as their homologous psychrophilic enzyme, or had less than 30% sequence identity.

**Table 1:** List of homologous enzymes used in this study

Psychrophilic Protein				Mesophilic Protein				
	**PDB**				**PDB**			
**Source Organism**	**Code**	**Resolution**	**Growth (°C)**	**Source Organism**	**Code**	**Resolution (Å) %**	**Identity**	**Classification**

*Salmo salar*	1a0j 1am	1.7	4	*Streptomyces griseus*	1sgt	1.7	33	Serine Protease
*Gadus morhua*	5	2.16	10	*Homo sapiens*	1qrp	1.96	59	Aspartyl Protease
*Alteromonas haloplanctis*	1aqh	2	4	*Sus scrofa*	1pif	2.3	46	Hydrolase
*Antarctic bacterium ds2-3r*	1a59	2.09	5	*Escherichia coli*	1k3p	2.2	30	Citrate Synthase
*Aquaspirillum arcticum*	1b8p	1.9	4	*Sus scrofa Acidaminococcus*	5mdh	2.4	50	Oxidoreductase
*Lactobacillus casei*	1dxy	1.9	15	*fermentans*	1 × dw	1.98	33	Oxidoreductase
*Salmo salar*	1elt	1.61	4	*Sus scrofa*	1eai	2.4	68	Serine Protease
*Pseudomonas sp.*	1g9k	1.96	10	*Pseudomonas aeruginosa*	1kap	1.64	50	Hydrolase
*Bacillus megaterium*	1gco	1.7	10	*Bacillus anthracis*	2uvd	2.4	38	Oxidoreductase
*Pandalus borealis*	1k7h	1.92	5	*Homo sapiens*	1zeb	1.9	41	Hydrolase
*Arthrobacter sp.*	1kfw	1.74	5	*Bacillus circulans*	litx	1.1	33	Hydrolase
*Lactobacillus brevis*	1n × q	1.79	15	*Mycobacterium tuberculosis*	lnff	1.8	40	Oxidoreductase
*Gadus morhua*	1okb	1.9	10	*Homo sapiens*	1akz	1.57	76	Hydrolase Fumarate
*Shewanella frigidimarina*	1qjd	1.8	20	*Shewanella putrefaciens*	1d4d	2.5	61	Reductase
*Bacillus globisporus*	1s3g	2.25	15	*Bacillus subtilis*	2ori	1.8	68	Transferase
*Vibrio sp. pa-44*	1sh7	1.84	19	*Bacillus pumilus*	1mee	2	39	Hydrolase
*Pseudoalteromonas*								
*haloplanktis*	1tvn	1.4	20	*Erwinia chrysanthemi*	1egz	2.3	63	Hydrolase
*Serratia sp.*	2b6n	1.8	4	*Bacillus lentus*	1ndu	1.6	37	Hydrolase
*Antarctic bacilli*	2gko	1.4	5	*Bacillus clausii ksm-k16 Chromobacterium*	1wsd	1.5	45	Hydrolase
*Colwellia psychrerythraea 34h*	2v27	1.5	10	*violaceum*	1ltu	1.74	34	Oxidoreductase

Dataset of 20 psychrophilic/mesophilic protein pairs, PDB code, species name, and structure resolution are provided.

### B-values

Psychrophilic enzymes sacrifice conformational stability to become more flexible, and with greater intrinsic flexibility they remain catalytically active at lower temperatures [11-13,15,17]. Nonetheless, the structural basis for this flexibility remains unclear. To investigate psychrophilic protein flexibility on a molecular level, we carried out a statistical comparison of crystallographic B-values between psychrophilic and homologous mesophilic proteins.

**Figure 1:**
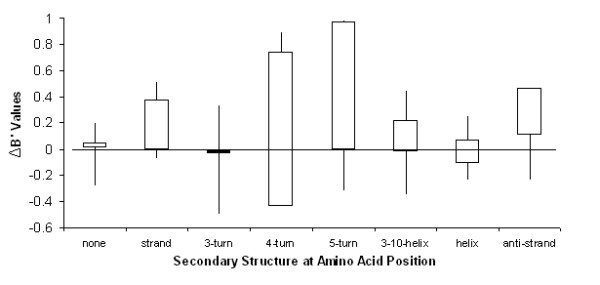
**ΔB'-value of each secondary structure**. Psychrophiles have greater atom flexibility in strands and 5-turns secondary structure (p < 0.001) compared to mesophilic proteins. * indicates outliers.

B-values are measurements of atomic fluctuations in protein crystals. Atomic fluctuations can be divided into dynamic motions and crystal lattice defects, but only the former is relevant to flexibility [23]. To isolate the atomic motion component from the lattice component, we first eliminated outliers from the raw B-values (an explanation of B-value outliers is given in the Methods section) and then normalized the resulting B-values based on the overall mean and standard deviation. This approach, which has been used previously [24] factors out overall differences by assuming the same overall distribution of atomic flexibility across proteins, allowing us to examine the regional distribution of B-value differences. Using the normalized B-values and a structure-based alignment, we calculated ΔB'_i _= B'_i_(psy)-B'_i_(mes) for all aligned positions *i*, as observed in Additional file 1, Figure S1 for the pair 1ELT and 1EAI. Relatively high values of ΔB'_i _indicate regions of higher flexibility in the psychrophilic protein relative to the mesophilic homolog. Distributions of ΔB'_i _across secondary structure as defined by the Dictionary of Protein Secondary Structure (DSSP) revealed interesting correlations (the number of total positions at each secondary structure is shown in Additional file 1, Figure S2). A plot of the calculated ΔB' values for each secondary structure can be seen in Figure 1 of the distribution of all 20 homolog pairs. The strand (p-value < 0.01) and the 5-turn (p-value < 0.01) secondary structures were significantly more flexible in psychrophilic proteins as compared to their mesophilic counterparts.

Following a similar methodology, Siglioccolo et al. [25] also analyzed B'-values of psychrophilic and mesophilic proteins grouped by common secondary structures (α-helices, β-sheets, and turns). They observed that β-sheets and turns tend to be more flexible in psychrophilic proteins, relative to helices. The results hold true for this new, larger set of homologous pairs. Because overall flexibility differences were factored out by normalization, we cannot distinguish an increased flexibility in β-sheets from a decreased flexibility in α-helices using this data; however, it is worth noting that β-sheets tend to sit in the core of a protein where lattice defects have less of an effect, leaving intrinsic flexibility as the likely cause of higher B-values.

### Cavity volume and morphology

B-values suggest, but do not inform, structural differences between a psychrophilic protein and its mesophilic counterpart, which prompted us to investigate the intrinsic characteristics of the protein core. Thermophilic globular proteins are known to have a highly compact hydrophobic core [3]. Hydrophobic packing in psychrophilic proteins has not been widely studied, but the increased presence of amino acids with smaller side-chains inside the protein, points to a weak hydrophobic core. The internal loose packing in the protein core relates to intrinsic flexibility, which has been previously noted [26].

**Figure 2:**
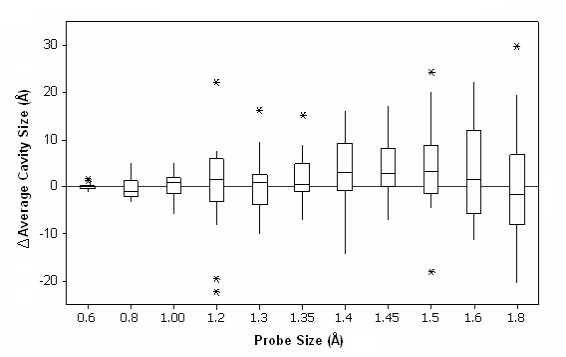
**Average size of cavities in psychrophilic proteins subtracted from the average sizes of the mesophilic cavities**. Positive ΔCavity Size indicates higher average cavity size in psychrophilic proteins at the indicated probe sizes. Red stars indicate statistically significant differences in average cavity size (p < 0.01). * indicates outliers.

Paired proteins were analyzed by comparing total void volume (pockets and cavities), total pocket volume (a pocket is a cavity that has a channel to the bulk solvent), total cavity volume, total number of voids (pockets and cavities), total number of pockets, and total number of cavities. The pockets and cavities were analyzed at probe radii ranging from 0.6 Å to 1.8 Å. The data was not normalized by molecular weight because for the most part each protein pair has similar molecular weights. Although it was expected that psychrophilic enzymes would have larger total cavity volumes when compared to their mesophilic counterparts, 40% of the psychrophilic proteins examined had smaller total cavity volumes throughout the entire range of probe sizes. Also, analysis of total number of cavities showed no significant differences between the two populations. No significant differences were found for either total void volume (pockets and cavities) or total pocket volume and number. However, a deeper analysis revealed a correlation wherein the average cavity volume is significantly larger (p < 0.01) in psychrophilic proteins at probe sizes equivalent to the size of a water molecule (1.4 to 1.5 Å). This was observed in two distinct ways: 1) when a psychrophilic enzyme had a smaller total cavity void volume than its mesophilic homolog, then the number of cavities in the former was less than in the latter, resulting in a larger average cavity volume in the psychrophilic enzyme; and 2) when a psychrophilic enzyme had more cavities than the homologous mesophilic enzyme, the total cavity volume was larger in the psychrophilic enzyme. Psychrophilic enzymes had an average cavity volume of 34 Å^3 ^compared to an average cavity volume of 30 Å^3 ^found in mesophilic enzymes at the 1.4 Å probe size. Figure 2 shows the distribution of the difference in cavity average size between each paired psychrophilic and mesophilic enzymes for all 20 pairs at different probe sizes. Our observation that cavities of the size of a water molecule are present in greater volume in psychrophilic enzymes, suggests that water may be bound to psychrophilic enzymes in greater numbers, enhancing the internal solvation.

### Amino acid frequency surrounding psychrophilic cavities

**Figure 3:**
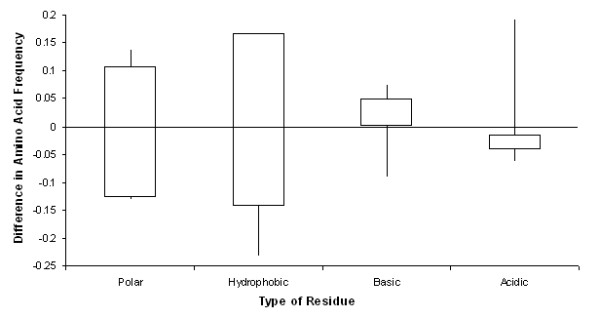
**Difference in frequency of each amino acid type on the inner cavity surface between psychrophilic and the mesophilic paired proteins**. Psychrophilic enzymes have a larger number of acidic side chains on the inner surface of a cavity (p < 0.01). Mesophilic enzymes have a larger number of hydrophobic residues (p < 0.01).

To better understand cavity composition, inward-facing side chains of amino acids that surround the cavities at probe size 1.4 Å were compared between psychrophilic and mesophilic proteins (Figure 3). Outward facing side chains were not considered because the backbone chemistry is the same in the two populations. Amino acids were classified into four groups: nonpolar, hydrophobic, basic, and acidic. It was observed that the average number of acidic amino acid residues lining psychrophilic protein cavities exceed the average number observed in homologous mesophilic protein cavities (p < 0.01). Moreover, there are fewer hydrophobic side chains surrounding psychrophilic cavities than mesophilic cavities (p < 0.01). Finally, cavities in psychrophilic proteins are more predominant in regions containing turns and coils, in contrast to mesophilic protein cavities (data not shown).

This study reveals that cavities in psychrophilic enzymes found at probe size 1.4 to 1.5 Å: (1) are statistically significantly different than in homologous mesophilic enzymes; and (2) their cavity surfaces contain a higher proportion of acidic amino acids. Several studies have found that cavities with these specific characteristics allow water molecules to exist stably within them [27-30]. Two key characteristics, in particular, are high polarity and excess space for movement. For example, Park and Saven [31] analyzed 6,718 buried water molecules contained in 842 different high resolution protein structures, revealing that these water molecules formed hydrogen bonds with polar atoms, predominantly near residues that compose turns or coils. Chen and Stites [4] obtained a similar result both experimentally and computationally with staphylococcal nuclease, wherein a water molecule stabilized Glu-66. These authors concluded that water molecules in polar cavities make more stable hydrogen bonds with the cavity walls and have longer residence times than in hydrophobic cavities. However, the presence of water and its function in protein cavities remains unclear.

It is well established that overall hydration increases the flexibility of the protein [26-30]. For example, Armstrong et al. [26], using electron paramagnetic resonance (EPR) spectroscopy, showed the importance of water hydration in the core of apomyoglobin and its role in protein transition between several structural conformational states, presumably by acting as a lubricant. The dynamics of water inside the protein core affects protein thermostability [26]. Another methodology used to understand the role of water in enzymes, specifically the role of water in catalysis, involves nonaqueous systems. Specifically, adding water to a final concentration of 1% (v/v) in tetrahydrofuran resulted in a significant increase in the proteolytic activity of subtilisin Carlsberg, concomitant with an increase in active site mobility as determined by EPR [32]. Using molecular dynamics simulations, Tarek and Tobias [33] demonstrated that higher levels of hydration contribute to increased in protein motion. Similarly, Rupley et al. [34], showed that a specific level of hydration is required by proteins to be active (0.4 g H_2_O per g dry protein). These findings point to an important correlation between hydration, protein flexibility, and enzymatic activity.

Many studies have proposed the importance of cavity hydration and its relation to higher protein flexibility. Fischer et al. [28] theoretically calculated the vibration entropy of bovine pancreatic trypsin inhibitor with bound and unbound water-122 (a buried water molecule). Bound water molecules increase the vibrational entropy of the protein, which could also be thought of as an increase in protein flexibility [28]. Garcia and Hummer [35] used MD simulations to observe that water molecules inside proteins slowly exchange with the solvent, and when the molecules escape or penetrate the protein, they cause dynamic fluctuations.

### Counting crystallographic waters

The observation that cavities in psychrophiles favor water-sized objects should imply that more waters are bound to the cold-adapted enzymes. To verify this, we isolated the buried crystallographic waters in all 38 enzymes (excluding the 1a59/1k3p pair because 1a59 contains no waters) by removing the surface-exposed waters and any waters that were buried by surface exposed waters, and then counting them. A correlation was observed (R^2 ^= 0.73) in the number of buried waters between psychrophilic and mesophilic homologs, showing a consistency across crystals and crystallographers in modeling ordered solvent (see Additional file 1, Figure S3). There is a visible trend towards more water molecules in psychrophilic enzymes; however, the significance is marginal (p = 0.05). Interestingly, counter examples in this set appear to possess large numbers of buried waters. In addition to 1a59, if we remove the five pairs of psychrophilic-mesophilic homologs with > 25 buried waters (i.e., those with a high overall water density that could skew the analysis), then 11 out of the remaining 14 pairs fit the trend of higher water content in psychrophilic enzymes than in mesophilic enzymes. Nonetheless, ignoring such data pruning, the difference is significant using either the binomial test (p = 0.03) or the paired t-test (p < 0.01). Moreover, we may not be counting all cavity water molecules because of possible experimental errors or water in cavities being highly mobile (especially in nonpolar cavities) [36]. Therefore, a more sensitive approach, e.g., MD simulations, should be considered to test the hypothesis that psychrophilic enzymes bind more water than their mesophilic counterparts.

## Conclusions

In this study we explored a variety of structural bioinformatic metrics to seek a structural explanation for cold adaptation in enzymes. The most significant structural differences are an increase in the size of the water-sized cavities and a trend in amino acid composition towards carboxylic acids in these cavities. Through an additional consensus of measures, including a significant increase in crystallographic B-values in **β**-sheets and turns, and a marginally significant increase in the number of buried crystallographic waters, we can conclude that psychrophilic enzymes tend to be more solvated in the core as compared to mesophilic enzymes. In particular, the evidence that psychrophilic cavities are well characterized by a water-sized probe suggests that mutations that reshaped internal cavities to fit water may have led to more bound water, which in turn led to an increased flexibility in the core, consistent with water-protein literature.

Statistical metrics suffer from small data sets, crystallographic variability, and the fact that multiple mechanisms for cold adaptation exist. Nonetheless, our results point to a common hypothesis that can now be tested by protein design experiments (e.g., increasing the number of acidic residues that comprise cavities). Cavities are not necessarily the only element of a protein that endows psychrophilic proteins with cold adaptation, but structure-based differences in cavities may reveal themselves to be critical to cold adaptation and might help to design enzymes capable of functioning at low temperatures. Similar results may be obtained in other rigidifying environments, such as organic solvents, polymeric materials [37,38], or protein-nanomaterial conjugate materials [39,40].

## Methods

### Database construction

The proteins used in this study were collected from the Protein Data Bank (PDB). The first set consisted of PDB structures of cold adapted enzymes proposed by Siddiqui et al. [12] as well as additional enzymes selected from the National Center of Biotechnology Information (NCBI) database (http://www.ncbi.nlm.nih.gov). To be considered, each protein was required to have at least 150 amino acids and a crystal structure with resolution better than 2.5 Å. To obtain valid mesophilic-psychrophilic homolog pairs, a series of steps were followed. First, DaliLite [41] (comparison of 3D structures) was used to obtain a set of homologous mesophilic proteins valid for comparison to their psychrophilic counterparts, using a requirement of at least 30% sequence identity. Proteins with resolution poorer than 2.5 Å were discarded. The Pfam [42] was used to confirm that each pair was homologous. Lastly, the Prokaryotic Growth Temperature Database (PGTdb) [43] was used to classify proteins by the growth temperature of the organism (psychrophiles 0-20°C, mesophiles 20-45°C, and thermophiles 45-100°C). Note that all eukaryotic organisms were mesophilic, with few exceptions.

### B-values

The crystallographic temperature factors (B-values) of alpha carbons were chosen as a measure of backbone flexibility. B-values vary greatly from protein to protein, due largely to differences in crystal quality and the idiosyncrasies of structural refinement. To compare psychrophilic and mesophilic proteins, and avoid uncertainties associated with proteins with high average or highly spread B-values, we used the method of Smith et al. [24]. Briefly, B-value outliers were detected and removed using a median-base method, indicated in Eq. 2.1, as follows:

(2.1) $$M_{i}=0.6745^{\prime }\left(X_{i}-X\right)∕MAD$$

Where X_i _is the B-value from the C-*α *carbon in the i^th ^residue, X is the median of the B-factors, and MAD is the median of absolute displacement. An M_i _> 3.5 is considered an outlier. Then after removal of outliers, we normalized B-factors using Eq. 2.2:

(2.2) $$B_{i}^{\prime }=\frac{B_{i}}{\sqrt{<B^{2}>}}$$

where B_i _is the B-value at the alpha carbon of the i^th ^sequence position, and <B^2^> is the average of (B_i_)^2 ^after removing outliers. After removing the outliers and normalizing the B-values in each protein, we compared B'-values between psychrophilic and mesophilic proteins in pairwise alignments, giving ΔB'_i _values.

(2.3) $$\Delta {\text{B}}_{\text{i}}^{\prime }=B_{\text{i}}^{\prime }\left(\text{psy}\right)-B_{\text{i}}^{\prime }\left(\text{mes}\right)$$

B'(psy)_i _is the normalized B-value of the psychrophile enzyme at the i^th ^position in a pairwise sequence alignment. Larger ΔB'-values imply a higher level of flexibility. Therefore, ΔB'_i _> 0 indicates that the psychrophilic protein is more flexible at that amino acid position than its homologous mesophilic protein.

### Cavity volumes

The Computed Atlas of Surface Topography of proteins (CASTp) [44] was used to test for the presence of cavities and the associated void volume. The program uses Delaunay triangulation and the alpha shapes algorithm to determine cavities and pockets. CASTp also outputs the set of amino acids surrounding each cavity/pocket, the cavity/pocket surface volume through solvent accessible surface calculation, and the number of exits from the pocket. A cavity is defined as a pocket with zero exits.

CASTp calculations were performed with the following probe radii: 0.6, 0.8, 1.0, 1.2, 1.4, 1.6 and 1.8 Å. In all cases, the goal was to identify cavities that were fully contained in the protein subunit and not at the interface between subunits. Crystallographic water molecules were ignored. For each protein pair the total number of cavities (Cav), total volume inside the cavities (using accessible surface) (Vol), and total volume in cavities divided by total number of cavities (Vol/Cav) were determined. A paired t-test was used to account for the correlation between the proteins pairs because they shared the same environment. The null hypothesis tested was that no differences were observed between the mean values of each population (psychrophilic and mesophilic proteins) with respect to the aforementioned measures.

### Amino acid frequency

Amino acid frequencies were calculated for inward-facing side chains surrounding the cavities in psychrophilic proteins, and were compared to those in mesophilic proteins. The amino acids were classified into four types: 1) hydrophobic (Ala, Cys, Ile, Met, Pro, Val, Leu, Phe, Trp); 2) polar (Asn, Gln, Gly, Ser, Thr); 3) basic (Arg, His, Lys); and 4) acidic (Glu, Asp), for analysis. Probability distributions over the four amino acid types were summed separately for psychrophiles and homologous mesophiles over cavity wall position. A paired two-sample t-test was used to assess the differences between each psychrophilic and mesophilic distribution. The p-value obtained from the paired two samples test is considered statistically significant when p-value < 0.01

### Ordered waters

The number of buried crystallographically ordered waters was determined by iteratively removing all waters with non-zero solvent exposed surface area, as determined by MASKER [45] until no further waters could be removed. A paired two-sample t-test and a binomial test were used to assess the differences between each psychrophilic and mesophilic distribution. The p-value obtained from the paired two samples test is considered statistically significant when p-value < 0.01. Approximately 20-30% of all cavities are unoccupied by water in both mesophiles and psychrophiles. This information suggests that at worst, there is an undercounting of the number of waters in cavities in both the psychrophiles and their homologous mesophiles, which should not significantly affect the statistics.

## Authors' contributions

JSD and CB designed the research project. DIP performed the analysis of the cavity data and B-factors. Data collection was carried out by KW and DIP. Pipeline program and scripts were written by DIP, DJP and CB. The manuscript was written and figures and tables prepared by JSD, CB, DIP, DJP and KW. All authors read and approved the final manuscript.

## Supplementary Material

Additional file 1 **Figure S1. ΔB'-values of psychrophilic serine protease (PDB:**1ELT**) and mesophilic serine protease (PDB: **1EAI**) at each amino acid position in a pairwise alignment**. Graphic of ΔB'-values from paired protein 1ELT and 1EAI. At the top is the secondary structure of the psychrophilic enzyme. One visible rigid region from (amino acids 1-110) and one flexible region (amino acids 111-235) are obtaining using the ΔB'-value methodology. **Figure S2. Table of number of positions found at each secondary structure in psychro/mesophilic pair**. Table of number of positions found at each secondary structure in the 20 psychro/mesophilic pairs. **Figure S3. Graphic of buried, crystallographic waters of each psychro/mesophilic pair**. Plot of buried crystallographic waters from the 20 psychro/mesophilic pairs. In cases where multiple chains were present in the crystal structure, the values were averaged. Note: 1a59 contains no reported crystallographic waters. Click here for file
