# Supplementary material for: Comparative void-volume analysis of psychrophilic and mesophilic enzymes: Structural bioinformatics of psychrophilic enzymes reveals sources of core flexibility
Source: BMC Struct Biol. 2011 Oct 20;11:42. doi: 10.1186/1472-6807-11-42 (PMC3224250; doi:10.1186/1472-6807-11-42)
Supplement: Additional file 1 — Figure S1. ΔB'-values of psychrophilic serine protease (PDB:1ELT) and mesophilic serine protease (PDB: 1EAI) at each amino acid position in a pairwise alignment. Graphic of ΔB'-values from paired protein 1ELT and 1EAI. At the top is the secondary structure of the psychrophilic enzyme. One visible rigid region from (amino acids 1-110) and one flexible region (amino acids 111-235) are obtaining using the ΔB'-value methodology. Figure S2. Table of number of positions found at each secondary structure in psychro/mesophilic pair. Table of number of positions found at each secondary structure in the 20 psychro/mesophilic pairs. Figure S3. Graphic of buried, crystallographic waters of each psychro/mesophilic pair. Plot of buried crystallographic waters from the 20 psychro/mesophilic pairs. In cases where multiple chains were present in the crystal structure, the values were averaged. Note: 1a59 contains no reported crystallographic waters. [file 1472-6807-11-42-S1.PDF]

Supplemental Information

Comparative Void-Volume Analysis of Psychrophilic and Mesophilic Enzymes: Structural bioinformatics of psychrophilic enzymes reveals sources of core flexibility

Diana I. Paredes[1], Kyle Watters[1], Derek Pitman[2], Chris Bystroff[2], and Jonathan S. Dordick[1,2,3§]

[1]Department of Chemical and Biological Engineering, Rensselaer Polytechnic Institute, Troy, NY, USA.

[2]Department of Biology, Rensselaer Polytechnic Institute, Troy, NY, USA.

[3]Department of Biomedical Engineering, Center for Biotechnology & Interdisciplinary Studies, Rensselaer Polytechnic Institute, Troy, NY, USA.

Additional File 1

Figure S1. ΔB-values of psychrophilic serine protease (PDB:1ELT) and mesophilic serine protease (PDB: 1EAI) at each amino acid position in a pairwise alignment.

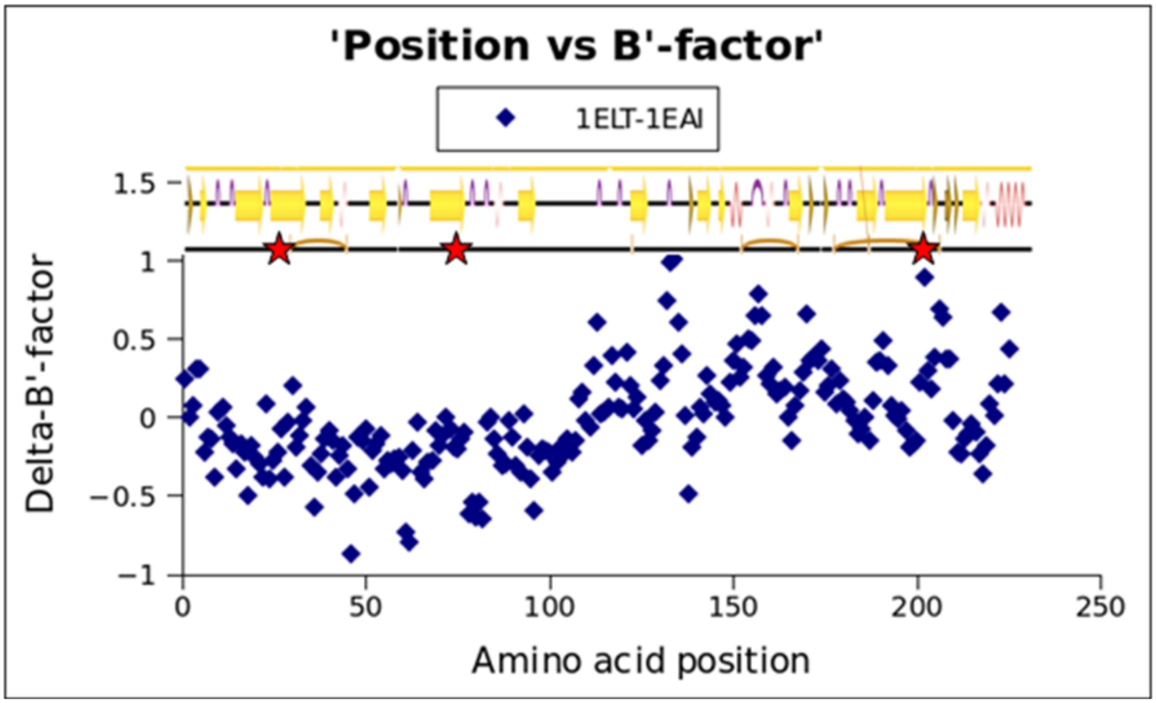

Description of data: Graphic of ΔB-values from paired protein 1ELT and 1EAI. At the top is the secondary structure of the psychrophilic enzyme. One visible rigid region from (amino acids 1-110) and one flexible region (amino acids 111-235) are obtaining using the ΔB'-value methodology.

Figure S2. Number of positions found at each secondary structure.

Protein Pairs		none	strand	3-turn	4-turn	5-turn	3-10-helix	helix
1SGT.A	1A0J.A	117	0	23	2	0	4	15
1K3P.A	1A59.A	89	0	23	0	5	10	183
1QRP.E	1AM5.A	79	26	21	8	8	13	29
1PIF.A	1AQH.A	158	36	30	4	4	43	98
5MDH.A	1B8P.A	67	27	23	3	3	13	144
1XDW.A	1DXY.A	99	47	31	3	5	8	130
1EAI.A	1ELT.A	95	0	31	0	2	10	15
1KAP.P	1G9K.A	191	76	49	3	2	23	63
2UVD.A	1GCO.A	72	22	17	0	4	15	99
1ZEB.A	1K7H.A	172	42	38	12	4	28	129
1ITX.A	1KFW.A	126	45	32	8	6	23	101
1NFF.A	1NXQ.A	55	37	22	3	2	9	96
1AKZ.A	1OKB.A	66	20	27	4	3	22	71
1D4D.A	1QJD.A	189	28	48	15	9	22	182
2ORI.A	1S3G.A	49	23	10	9	3	2	104
1SUP.A	1SH7.A	89	37	26	8	1	3	69
1EGZ.A	1TVN.A	76	44	22	3	4	6	104
1EA7.A	1V6C.A	101	36	27	8	2	3	67
1MEE.A	2B6N.A	86	37	24	6	2	4	71
1WSD.A	2GKO.A	91	36	26	6	4	1	74
1LTU.A	2V27.A	68	14	8	6	2	12	118
**Total**		**2135**	**633**	**558**	**111**	**75**	**274**	**1962**

Figure S3. Graphic of buried, crystallographic waters of each psychro/mesophilic pair.

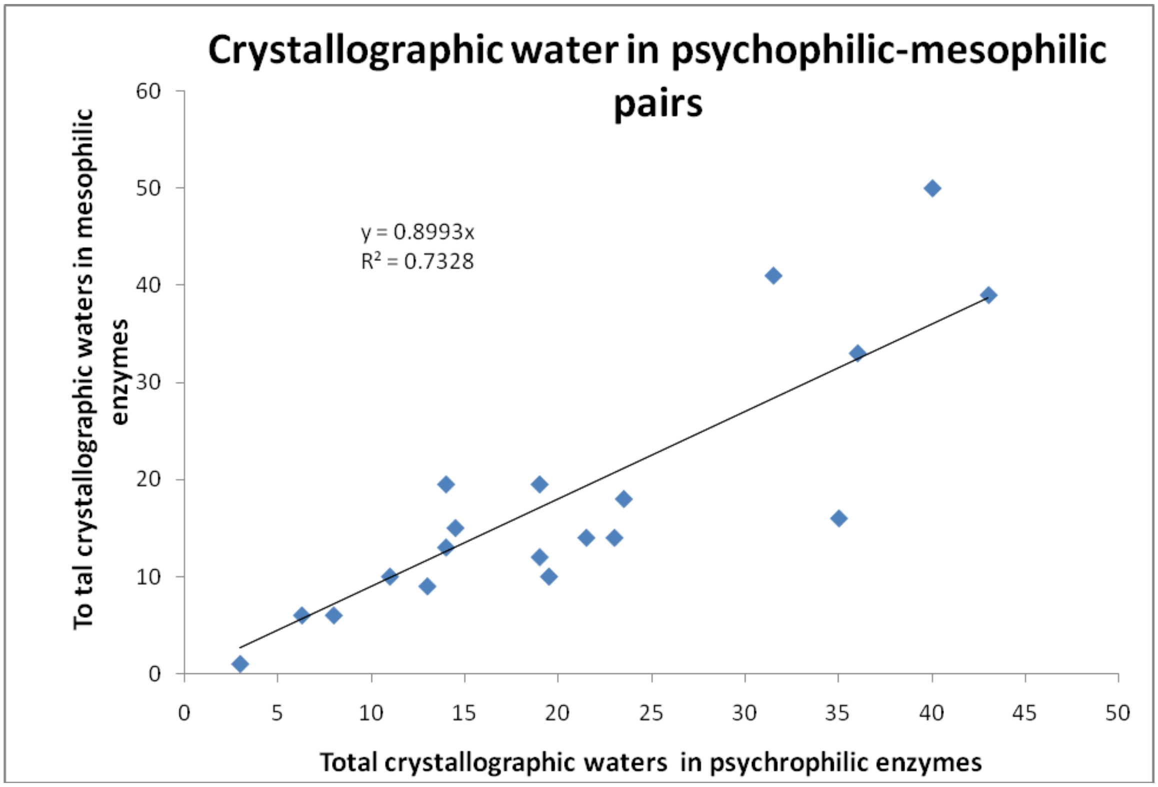

Description of data: Plot of buried crystallographic waters from the 20 psychro/mesophilic pairs. In cases where multiple chains were present in the crystal structure, the values were averaged. Note: 1a59 contains no reported crystallographic waters.
